# Nestin as a Marker Beyond Angiogenesis—Expression Pattern in Haemangiomas and Lymphangiomas

**DOI:** 10.3390/biomedicines13030565

**Published:** 2025-02-24

**Authors:** Andreas Mamilos, Lina Winter, Christoph B. Wiedenroth, Tanja Niedermair, Stefanie Zimmer, Volker H. Schmitt, Karsten Keller, Ondrej Topolčan, Marie Karlíková, Markus Rupp, Christoph Brochhausen, Cristina Cotarelo

**Affiliations:** 1 Institute of Pathology, University Regensburg, 93053 Regensburg, Germany; 2 Department of Pathology, German Oncology Centre, 4108 Limassol, Cyprus; 3 Medical Faculty, European University of Cyprus, 2404 Nicosia, Cyprus; 4 Institute of Pathology, Medical Faculty Mannheim, Heidelberg University, 68167 Mannheim, Germany; 5 Department of Thoracic Surgery, Kerckhoff Klinik, 61231 Bad Nauheim, Germany; 6 Institute of Pathology and Tissue Bank, University Medical Center Mainz, 55131 Mainz, Germany; 7 Department of Cardiology, University Medical Center of the Johannes Gutenberg-University Mainz, 55131 Mainz, Germany; 8 German Center for Cardiovascular Research (DZHK), Partner Site Rhine Main, 55131 Mainz, Germany; 9 Center for Thrombosis and Hemostasis (CTH), University Medical Center of the Johannes Gutenberg-University Mainz, 55131 Mainz, Germany; 10 Medical Clinic VII, Department of Sports Medicine, University Hospital Heidelberg, 69120 Heidelberg, Germany; 11 Central Laboratory for Immunoanalysis, Faculty of Medicine, Pilsen Charles University, 323 00 Pilsen, Czech Republic; 12 Department for Trauma Surgery, University Hospital Regensburg, 93053 Regensburg, Germany

**Keywords:** nestin, haemangioma, lymphangioma, endothelial cells, immunohistochemistry, histology, CD31, D2-40

## Abstract

**Background**: The intermediate filament nestin was first described in stem and progenitor cells of neural and mesenchymal origin. Additionally, it is expressed in endothelial cells during wound healing and tumorigenesis. Thus, nestin is widely regarded as a marker for proliferative endothelium. However, little is known about its role in lymphatic endothelium. **Methods**: Here, we analyzed the expression of nestin in the endothelium of ten human haemangiomas and ten lymphangiomas in situ by immunohistochemistry. This study aimed to investigate the expression of nestin in haemangiomas and lymphangiomas to determine its potential role as a vascular marker. Specifically, we aimed to assess whether nestin expression is restricted to proliferating endothelial cells or also present in non-proliferative blood vessels. **Results**: Immunohistochemically, haemangiomas were positive for CD31 but negative for D2-40. The endothelial cells within these lesions showed a homogeneous expression of nestin. In contrast, the endothelium of lymphangiomas reacted positively for D2-40 and CD31 but did not show any nestin expression. Additionally, only a few endothelial cells of capillary haemangiomas showed a Ki-67 positivity. **Conclusions**: The differential expression of nestin in haemangiomas and lymphangiomas indicates a specificity of nestin for the endothelium of blood vessels. The Ki-67 negativity in the majority of the endothelial cells reveals the proliferative quiescence of these cells. These findings indicate that nestin could be used as a marker to differentiate between blood and lymphatic vessels.

## 1. Introduction

Since its first description in 1990 by Lendahl et al., the intermediate filament (IF) protein nestin was assumed as a marker for precursor or stem cells, especially of neuroectodermal and mesenchymal origin [1,2,3]. It is initially expressed during the early development and is later downregulated and replaced by tissue-specific IF proteins such as neurofilaments in neurons and desmin in muscle cells [4,5]. Nestin requires interaction with other IF proteins for assembly and plays a crucial role in mitosis by regulating vimentin bundle disassembly [6,7,8]. Thus, nestin seems to play a role in the distribution of IF proteins to the daughter cells in dividing cells [6].

Initially considered specific to several stem or progenitor cells, nestin is now associated with activated, proliferating cells in both physiological and pathological conditions [9,10,11]. For instance, its expression has been observed in tissue regeneration, including ischemic myocardium [12], acute renal ischemia [13], pulmonary fibrosis [14], and wound healing [15,16]. Furthermore, its presence in diverse tumor types correlates with varying prognostic outcomes, indicating its potential as a biomarker [17,18,19]. Beyond tumor angiogenesis, nestin is expressed in physiological angiogenesis, such as in the pancreas [20], skin [21], and corpus luteum [22]. These findings were confirmed by in vitro analyses showing the specific expression of nestin in proliferative endothelial cells and endothelial progenitor cells, whereas mature endothelial cells lack nestin expression [23,24,25].

Although lymphatic endothelial cells derive from blood endothelial cells during embryogenesis, there exhibit distinct molecular characteristics, including differences in their development and in the expression of specific markers [26,27,28]. In contrast to the well-studied role of nestin in endothelial cells of blood vessels, little is known about its expression in the lymphatic system. Up to now, it has only been described in developing lymph nodes and fibroblastic reticular cells in the adult lymph nodes [29].

Therefore, the aim of this study was to systematically investigate the expression of nestin in blood and lymphatic endothelial cells to gain new insights into its role in human vascular biology. In the present report, we used human haemangiomas and lymphangiomas, which are benign vascular lesions lined by a monolayer of endothelial cells, as models to study the nestin expression in blood and lymphatic vessels in situ. In contrast to previous analyses that primarily considered nestin as a marker for proliferating endothelial cells and neoangiogenesis, we aimed to determine whether its expression is also present in non-proliferating blood vessels.

## 2. Materials and Methods

### 2.1. Study Design

This study follows an observational design with a comparative immunohistochemical analysis of haemangiomas and lymphangiomas to investigate nestin expression in vascular and lymphatic cells. This study was approved by the ethical review board of the University of Regensburg.

### 2.2. Tissue Samples

Human tissue samples from surgically resected haemangiomas (n = 10; age range 6–88 years; median age 50 years) and lymphangiomas (n = 10; age range 26–81 years; median age 53.5 years) were obtained retrospectively from the archives of the institute of pathology. Inclusion criteria comprised histopathologically confirmed haemangiomas and lymphangiomas with available formalin-fixed, paraffin-embedded (FFPE) tissue samples. Only surgically resected lesions with sufficient endothelial representation and quality were included. The age of the patients was no relevant inclusion criterion. Surgical removal of haemangiomas and lymphangiomas was performed due to clinical indications such as lesion growth, symptomatic presentation (e.g., pain, bleeding, or functional impairment), cosmetic concerns, or diagnostic uncertainty requiring histopathological confirmation.

### 2.3. Histological and Immunohistological Staining

The samples were fixed in 4% buffered formalin, processed routinely according to standardized methods, and embedded in paraffin. For histomorphological examination, 3–4 µm thick tissue sections were performed and stained with hematoxylin and eosin in a fully automated manner (Leica ST4040; Leica, Wetzlar, Germany). Following the peroxidase method, immunohistochemical analyses were performed according to standardized protocols using a Techmate 500 plus autostainer (Dako, Glostrup, Denmark). The 3–4 µm thick tissue sections were deparaffinized and rehydrated. For antigen retrieval, the samples were treated in citrate buffer with a steamer (Gourmet Plus; Braun, Kronberg, Germany) for 15 min. Sections—to be later stained with antibodies against CD31—were treated with microwave (600 W, 3 × 5 min) and trypsin (0.01% for 15 min) instead of a steamer. The following primary antibodies were used: mouse monoclonal anti-nestin (Chemikon, Temecula, CA, USA); mouse monoclonal anti-D2-40 (Signet, Dedham, MA, USA); mouse monoclonal anti-CD31 (Dako, Glostrup, Denmark); mouse monoclonal anti-Ki-67 (Dako, Glostrup, Denmark). Characteristics of the used primary antibodies are summarized in Table 1. After incubation with a biotinylated secondary antibody and streptavidin-coupled peroxidase, sections were stained with 3,3′ Diaminobenzidine (DAB), and nuclei were counterstained with hematoxylin.

### 2.4. Immunohistochemical Analyses

Slides were evaluated with a BX45 light microscope (Olympus, Tokyo, Japan), and the results were scanned with the PreciPoint M8 Microscope and Scanner (PreciPoint, Garching, Germany). Staining was semiquantitatively evaluated and scored by two independent investigators as follows: 0 = no positive vessels/endothelial cells; 1 = less than 10% positive vessels/endothelial cells; 2 = 10 to 50% positive vessels/endothelial cells; 3 = more than 50% positive vessels/endothelial cells.

Staining of podocytes in human kidneys was used as a positive control for nestin antibody. For CD31 and D2-40, staining of blood vessels and lymphatic vessels in the surrounding tissue served as positive controls. In cases of subcutaneous lesions, the basal cells of the epidermis served as an internal positive control for Ki-67. Additionally, negative controls without the use of first antibodies were performed.

## 3. Results

### 3.1. Histomorphological Results

All cases of haemangiomas showed typical morphological features: four cases of cavernous haemangiomas were composed of large, cystically dilated vessels with thin vessel walls lined by a layer of flat endothelial cells. In five cases, the lesions were made up of small capillary-like vessels with a lobular growth pattern, which gave evidence for the diagnosis of capillary haemangiomas. One case showed a mixed morphology with partly capillary, partly cavernous dilated vessels, classified as mixed capillary-cavernous haemangioma (Table 2).

**Figure 1:**
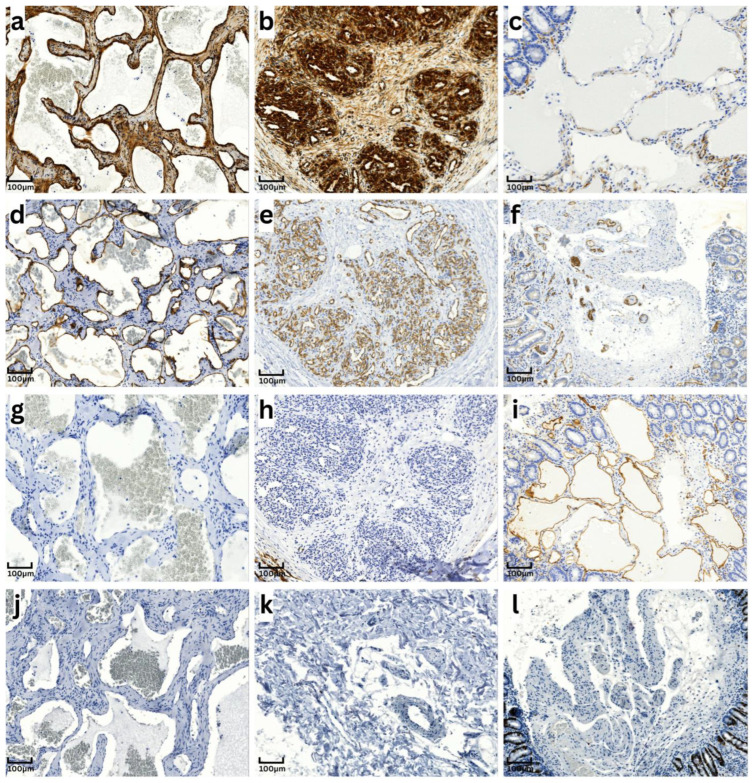
Immunohistological results in cavernous and capillary haemangiomas. Cavernous haemangiomas are shown in the left column, capillary haemangiomas in the middle column and lymphangiomas in the right column. (**a**–**c**) nestin staining; (**d**–**f**) CD31 staining; (**g**–**i**) D2-40 staining; (**j**–**l**) Ki-67 staining, 40× magnification.

Lymphangiomas were composed of dilated vessel convolutes with thin vessel walls and occasionally filled with a number of lymphatic cells. Within the lesions, immunohistochemistry revealed positivity of endothelial cells for the lymphatic marker D2-40 (Figure 1i), confirming the diagnosis of lymphangiomas.

### 3.2. Immunohistochemical Results

The scores of the different immunohistochemical stains are summarized in Table 2 for haemangiomas and Table 3 for lymphangiomas.

Nestin expression was detected in the endothelial cells in all cases of both cavernous (Figure 1a) and capillary haemangiomas (Figure 1b), as well as the one mixed haemangioma. In most cases, a strong expression could be found. Furthermore, the blood vessels in the surrounding tissue showed a homogeneous nestin expression. In contrast, endothelial cells in nine out of ten lymphangiomas were completely nestin-negative (Figure 1c). In only one case, a minor percentage of the endothelial cells (10–50%) showed weak expression. However, all blood vessels in the surrounding tissue of these lesions demonstrated a homogeneous nestin expression. Thus, nestin-positive blood vessels served as an internal positive control for the nestin staining in lymphangiomas.

Expression of the panendothelial marker CD31 were detected in endothelial cells in all haemangiomas and lymphangiomas, including the vessels in the surrounding tissue (Figure 1d–f). However, the lymphatic endothelial cells in lymphangiomas and lymphatic vessels in the surrounding tissue in all analyzed cases showed a slight discontinuity in the expression of CD31.

D2-40 is a specific marker for lymphatic endothelium and was detected in all cases of lymphangiomas (Figure 1i). In contrast, the endothelial cells in all cases of haemangiomas revealed no D2-40 expression (Figure 1g,h). As internal positive control, the endothelial cells in lymphatic vessels in the surrounding tissue in all examined specimens reacted positively with D2-40. Additionally, these D2-40 positive lymphatic vessels were negative for nestin.

In four of the ten cases of haemangiomas, a positive reaction with antibodies against the proliferation marker Ki-67 was found in less than 10% of the endothelial cells (Figure 1k). Three of these cases were capillary haemangiomas, and one was a mixed capillary-cavernous haemangioma. The other six cases, two capillary haemangiomas, and all four cavernous ones, were completely negative for Ki-67 (Figure 1j). The endothelium of the lymphangiomas revealed no expression of Ki 67 in any of the analyzed cases (Figure 1l). Endothelial cells in both blood and lymphatic vessels in the surrounding tissue were negative for Ki-67, indicating no proliferative potential of the endothelium. In cases of subcutaneous haemangiomas or lymphangiomas, respectively, the basal cells of the epidermis showed Ki-67 expression and served as internal control for this antibody.

## 4. Discussion

The present study revealed that nestin is expressed in haemangiomas, but not in lymphangiomas. Furthermore, we demonstrated that the expression of nestin is not exclusive to endothelial progenitor cells, as was previously assumed, but can also be identified in mature blood vessel endothelium. These results suggest that nestin plays not only a role in blood vessel formation, but also in non-proliferative blood vessels.

The type VI intermediate filament nestin has been detected in several cell types, especially in precursor or stem cells and proliferating cells [1,2], but also in several different tumors [17,18]. Until today, endothelium nestin expression has been predominantly shown in proliferating cells and regenerating tissue but not in the endothelial cells of mature blood vessels [9,10,25,30]. This led to the conclusion that nestin could serve as a marker for angiogenesis or neovascularization, respectively. In contrast, the role of nestin in endothelial cells of lymphatic vessels has not yet been characterized in detail. Therefore, we compared nestin expression in endothelial cells of haemangiomas and lymphangiomas, which are benign vascular tumors of different origins. Previously, the expression rate of nestin was shown to be higher in capillary haemangiomas than in malignant vascular tumors [31]. Other studies reported nestin expression only in capillary but not in cavernous haemangiomas [32,33]. In contrast, we clearly demonstrated a homogeneous nestin expression in the complete endothelium of both capillary and cavernous haemangiomas. Furthermore, the blood vessels in the surrounding tissue of all analyzed cases were also positive for nestin. Additionally, in haemangiomas, all nestin-positive vessels were also positive for CD31 but negative for D2-40.

Contrary to its relatively high expression in haemangiomas, almost no nestin expression was detected in the endothelium of the analyzed lymphangioma samples. In only one case, a small portion of endothelial cells (between 10 and 50%) within the lesion was stained positive for nestin implying the diagnosis of a haematolymphangioma. These results are consistent with a previous study showing that nestin is not expressed in lymphatic endothelial cells in lymph nodes [29]. In conclusion, lymphatic endothelial cells do not express nestin in general, which demonstrate the phenotypic differences in endothelial cells in lymphatic and blood vessels. Furthermore, the pan endothelial marker CD31 was found in most lymphatic endothelial cells with a slight discontinuity, confirming previous findings showing the discontinuity of several endothelial markers in lymphatic endothelial cells [34]. Taking our findings together, the marked difference in nestin expression in lymphatic and blood vessels underlines the differences in endothelial cells in the two branches of the vasculature. These findings may have relevance for routine histopathological diagnostics in some specific aspects, since, in addition to lymph vessels, serosal mesothelium also physiologically expresses D2-40 [35]. Thus, in the histopathological evaluation nestin may serve as an additional marker to distinguish cells and lesions, respectively, between mesothelial origin or lymphatic origin as, for example, in serosal inclusion cysts.

In the present collective, in only four cases of haemangiomas (three capillary and one mixed capillary-cavernous), we found a slight (less than 10%) expression of Ki-67 in the endothelial cells within the lesions. Abe et al. reported a Ki-67 index of 7.2 ± 3.7% in cutaneous capillary haemangioma patients with a median age 51 years (13–76 years) [36]. Furthermore, previous reports showed that Ki-67 is rarely expressed in haemangiomas, in general, but is significantly higher in capillary haemangiomas compared to cavernous ones [37,38]. Since Ki-67 detects cells in all phases of the cell cycle except those in G0-phase [39], the negativity for this marker indicates that those cells are proliferatively quiescent. Thus, Ki-67 negativity implies an absolute resting state of the cell.

Therefore, our findings deny the conventional assumption of nestin being specifically expressed in proliferative endothelium, endothelial progenitor cells, or during angiogenesis and suggest that nestin takes a broader role in vascular homeostasis. Correspondingly, Dusart et al. demonstrated through transcriptomic and proteomic analyses that nestin is expressed in endothelial cells across multiple vascular beds, independent of the proliferative status. Furthermore, their study showed that siRNA-mediated knockdown of nestin led to increased endothelial proliferation. This suggests that nestin plays a regulatory role in maintaining vascular stability, rather than simply serving as a marker of angiogenic activity [40]. These findings, together with our histopathological observations, emphasize the need for further functional studies to elucidate nestin’s precise role in vascular remodeling, endothelial plasticity, and the response to physiological stressors such as shear forces and hypoxia.

Previous studies have linked nestin to endothelial activation during tissue regeneration and tumor angiogenesis [41,42]. Taking the results of the present study together with previous findings, a reevaluation of nestin’s role in tumor angiogenesis may be needed. In this context, it is important to mention that, until now, nestin-positive tumor blood vessels are interpreted as angiogenic blood vessels. Since tumors contain a mixture of proliferating, remodeling, and stabilized vessels, nestin may influence vascular maturation beyond proliferation [43]. Future analyses might take our findings into consideration when investigating the tumor microenvironment and its vascularization.

The present study has several limitations. The small sample size restricts the generalizability of the results and limits the statistical power. A future study with a larger cohort would be beneficial to confirm the findings. Furthermore, the study does not investigate the molecular signaling pathways associated with nestin expression. Future research using RNA sequencing, protein interaction studies, and pathway analyses could help to determine nestin’s functional role in endothelial cells. Lastly, potential confounding factors such as patient-specific variables (e.g., age, comorbidities, genetic background) or differences in lesion location and microenvironment (e.g., hypoxia, inflammation) were not specifically controlled our study. These factors may influence nestin expression and should be addressed in future research to improve the robustness of the findings.

However, despite these limitations, this study provides a valuable contribution to the field by offering new insights into nestin expression in vascular and lymphatic endothelial cells.

## 5. Conclusions

In conclusion, our findings clearly demonstrate that nestin expression is not limited to endothelial progenitor cells but was also detected in the endothelium of non-proliferative, Ki-67 negative blood vessels. Thus, these findings underline nestin as an endothelial marker for blood vessels, which cannot distinguish between the functional status such as senescence, neo-angiogenesis or proliferation, respectively. In contrast, lymphatic endothelial cells do not express nestin. Therefore, nestin may be a valuable marker for vascular endothelial cells and could be used as an alternative for D2-40 in the discrimination of lymphatic from blood vessels.

## Figures and Tables

**Table 1:** Data on the primary antibodies used in the present study.

Antibody	Source	Host	Clone	Dilution	Pretreatment
Nestin	Chemikon, Temecula, CA, USA	Mouse	10C2	1:200	Steamer + citrate buffer
CD31	Dako, Glostrup, Denmark	Mouse	JC70A	1:50	Microwave + trypsin
D2-40	Signet, Dedham, MA, USA	Mouse	D2-40	1:50	Steamer + citrate buffer
Ki-67	Dako, Glostrup, Denmark	Mouse	MIB-1	1:400	Steamer + citrate buffer

**Table 2:** Characteristics of haemangiomas and scoring of the immunohistochemical staining.

Age	Location	Type	Nestin	CD31	D2-40	Ki-67
15	testis	cavernous	2	3	0	0
60	umbilicus	capillary	3	3	0	0
37	upper arm	capillary	3	3	0	0
6	back	capillary	3	3	0	1
72	finger	cavernous	3	3	0	0
59	liver	cavernous	3	3	0	0
37	thigh	cavernous	3	3	0	0
48	lip	mixed	3	3	0	1
88	palpebra	capillary	3	3	0	1
52	hand	capillary	3	3	0	1

Scoring: 0 = 0% positive cells; 1 = <10% positive cells; 2 = 10–50% positive cells; 3 = >50% positive cells.

**Table 3:** Characteristics of lymphangiomas and scoring of the immunohistochemical staining.

Age	Location	Nestin	CD31	D2-40	Ki-67
26	lip	0	3	3	0
81	duodenum	0	3	3	0
67	duodenum	0	2	3	0
68	mesocolon	0	3	3	0
27	not specified	2	3	2	0
73	kidney	0	2	2	0
42	abdominal wall	0	3	3	0
44	pancreas	0	2	3	0
46	supraclavicular	0	2	3	0
61	paraaortal	0	3	2	0

Scoring: 0 = 0% positive cells; 1 = <10% positive cells; 2 = 10–50% positive cells; 3 = >50% positive cells).

## Data Availability

Data available on request due to legal restrictions.

## Abbreviations

The following abbreviations are used in this manuscript:

DAB	3,3′-Diaminobenzidine
FFPE	formalin-fixed, paraffin-embedded
IF	intermediate filament
